# Situs inversus totalis with perforated duodenal ulcer: a case report

**DOI:** 10.1186/1752-1947-5-279

**Published:** 2011-07-03

**Authors:** Mohammad Tayeb, Faiz Mohammad Khan, Fozia Rauf

**Affiliations:** 1 Department of Surgery, Peshawar Medical College, Peshawar, Pakistan; 2 Department of Pathology, Peshawar Medical College, Peshawar, Pakistan

## Abstract

**Introduction:**

Situs inversus is an uncommon anomaly. Situs inversus viscerum can be either total or partial. Total situs inversus, also termed as mirror image dextrocardia, is characterized by a heart on the right side of the midline while the liver and the gall bladder are on the left side. Patients are usually asymptomatic and have a normal lifespan. The exact etiology is unknown but an autosomal recessive mode of inheritance has been speculated. The first case of perforated duodenal ulcer with situs inversus was reported in 1986; here, we report the second case of this nature in the medical literature.

**Case presentation:**

A 22-year-old Pakistani man presented with severe epigastric and left hypochondrial pain. Examination and investigations (chest X-ray and ultrasonography) confirm peritonitis in a case of situs inversus totalis. On exploratory laparotomy, a diagnosis of situs inversus totalis with perforated duodenal ulcer was confirmed. Graham's patch closure of the duodenal ulcer was performed with absorbable sutures, and a thorough peritoneal lavage was also performed; an incidental appendectomy was also performed to avoid further diagnostic problems. Our patient had an uneventful recovery.

**Conclusions:**

A diagnostic dilemma arises whenever abdominal pathology occurs in patients with situs inversus. Although an uncommon anomaly, to choose a proper surgical incision site for abdominal exploration pre-operative recognition of the condition is important.

## Introduction

Situs inversus, first described by Aristotle in animals and Fabricius in humans [1], is an uncommon anomaly with an incidence varying from one in 4,000 to one in 20,000 live births [2]. Situs inversus viscerum can be either total or partial. Total situs inversus, also termed as mirror image dextrocardia, is characterized by a heart on the right side of the midline while the liver and the gall bladder are on the left side. Patients are usually asymptomatic and have a normal lifespan. The exact etiology is unknown but an autosomal recessive mode of inheritance has been speculated [3]. However, situs inversus abdominus, characterized by 'mirror image' of the normal bowel, is caused by a clockwise rotation of the viscera during early embryonic life [4]. Very few cases of situs inversus totalis have been described in the literature.

## Case presentation

**Figure 1:**
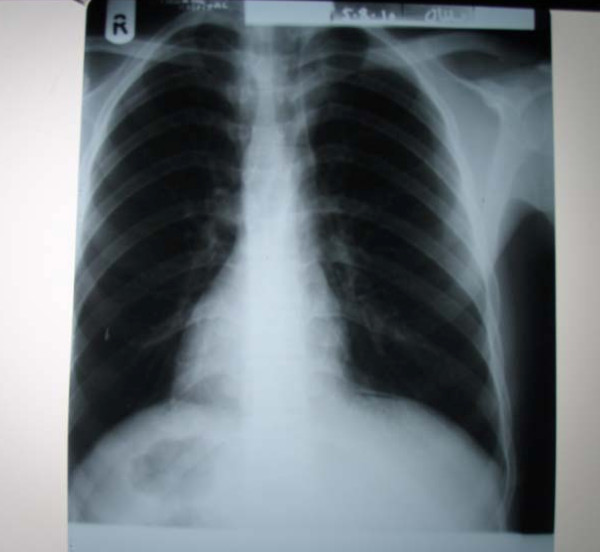
**X-ray of the chest taken in the erect position, showing dextrocardia, fundic gas shadow under the right dome of the diaphragm, the liver shadow and free gas under the left dome of diaphragm**.

A 22-year-old Pakistani man, who was a smoker and hashish user, was admitted to the emergency department of our hospital with sudden onset of severe epigastric and left hypochondrial pain for last 12 hours. He also complained of nausea and vomiting. He had a history of recurrent episodes of epigastric and left hypochondrial pain. A physical examination revealed a pulse rate of 105 beats/minute, blood pressure of 110/70 mmHg, and he was afebrile. Examination of his abdomen revealed guarding and rigidity, especially in the epigastrium and left hypochondrium. The laboratory results showed a serum hemoglobin level of 11 g% and a white cell count of 16,000 cmm with neutrophilia. His serum amylase level was at the upper limit of normal, but other biochemical test results were essentially normal. Results of an X-ray of the chest taken in the erect position showed dextrocardia, a fundic gas shadow under the right dome of diaphragm and a liver shadow on the left side. There was free gas under the left dome of the diaphragm (Figure 1). A clinical diagnosis of perforated duodenal ulcer in a case of dextrocardia with situs inversus was made. An electrocardiogram performed subsequently was diagnostic of dextrocardia with no other abnormalities. Ultrasonography confirmed the suspicion of situs inversus by demonstrating the presence of a left-sided liver and a left-sided normal gall bladder without any calculi. The spleen was on the right side with normal echotexture.

Our patient was unaware of this condition until this point. The brother of our patient was a doctor, who informed us that their paternal grandfather also had situs inversus totalis that had been diagnosed incidentally during an ultrasonography performed for prostatic symptoms; he was living a normal life.

After resuscitation with intravenous fluids, antibiotics, omeprazole, analgesics and nasogastric aspiration, our patient was subjected to an exploratory laparotomy. The diagnosis of perforated duodenal ulcer was confirmed. There was acute perforation of about 5 mm diameter in the anterior wall of the first part of the duodenum. There was complete situs inversus 'mirror image', with the liver and gall bladder on the left side and spleen on the right side. The stomach fundus was on the right and the first part of the duodenum lying to the left of the midline in the left hypochondrium. Exploration of the rest of the abdomen showed features typical of situs inversus totalis, that is, the caecum and appendix in the left iliac fossa and the sigmoid colon on the right.

A Graham's patch closure of the duodenal ulcer was performed with absorbable sutures, and a thorough peritoneal lavage was performed; an incidental appendectomy was also performed to avoid further diagnostic problems and the abdomen was closed in layers. Our patient had an uneventful recovery. Post-operatively he was counseled about cessation of smoking and hashish and was sent home on omeprazole therapy.

## Discussion

Situs inversus abdominus is an uncommon anomaly with an incidence varying from one in 4,000 to one in 20,000 live births [2]. Situs inversus usually remains undiagnosed, as exemplified by the present case, unless it is diagnosed incidentally while investigating another associated ailment. A diagnostic dilemma arises whenever pathology occurs in the unusual located abdominal viscera. To choose a proper surgical incision for abdominal exploration, pre-operative recognition of the condition is important. In our case the diagnosis was made pre-operatively and an exploratory laparotomy was performed with an upper midline incision.

Certain congenital anomalies such as polysplenia, asplenia or Kartagener's syndrome are known to occur in such patients [5,6]. However, our patient did not have any of these abnormalities.

Various modalities such as electrocardiograms, radiographic studies, computed tomography (CT) scans with oral and intravenous contrast, ultrasound, and barium studies can be used to diagnose situs inversus [7,8]. In our case, we diagnosed the condition by a chest radiograph and abdominal ultrasonography.

There have been isolated reports of situs inversus associated with peptic ulcer [9], ulcer perforation [10], amoebic liver abscess [11], acute cholecystitis [12], cholelithiasis [13,14], acute appendicitis [15], and intestinal obstruction [16]. To the best of our knowledge, this is only the second report in the literature of a patient with situs inversus totalis presenting with perforated duodenal ulcer (Gandhi *et al. *reported the first case of perforated duodenal ulcer with situs inversus in 1986 [10]).

## Conclusions

A diagnostic dilemma arises whenever abdominal pathology occurs in patients with situs inversus. Although an uncommon anomaly, to choose a proper surgical incision site for abdominal exploration pre-operative recognition of the condition is important.

## Consent

Written informed consent was obtained from the patient for publication of this case report and any accompanying images. A copy of the written consent is available for review by the Editor-in-Chief of this journal.

## Competing interests

The authors declare that they have no competing interests.

## Authors' contributions

MT performed the surgery and wrote the main part of the manuscript. FMK and FR reviewed the manuscript and made valuable changes.
